# Supplementary material for: MetaRibo-Seq measures translation in microbiomes
Source: Nat Commun. 2020 Jun 29;11:3268. doi: 10.1038/s41467-020-17081-z (PMC7324362; doi:10.1038/s41467-020-17081-z)
Supplement: Supplementary file 10 — Supplementary Data 7 [file 41467_2020_17081_MOESM10_ESM.zip › File2/Confidence_High_Taxonomy/109759_out.krona.html]

Javascript must be enabled to view this page.

members
magnitude
magnitudeUnassigned
count
unassigned
taxon
rank

109759\_out

2


SRS013687\_contig\_number\_contig-100\_13449.134162
1

superkingdom
2
1

1
1239
phylum

class
186801
1

order
1
186802

family
541000
1

genus
1
216851

1
1946503

SRS051031\_contig\_number\_5112
species
